# Supplementary material for: Senescent Human Pancreatic Stellate Cells Secrete CXCR2 Agonist CXCLs to Promote Proliferation and Migration of Human Pancreatic Cancer AsPC-1 and MIAPaCa-2 Cell Lines
Source: Int J Mol Sci. 2022 Aug 17;23(16):9275. doi: 10.3390/ijms23169275 (PMC9409091; doi:10.3390/ijms23169275)
Supplement: Supplementary file 1 [file ijms-23-09275-s001.zip › ijms-1831355-supplementary.pdf]

Supplementary Table S1. Gene list involved in Gene Ontology (GO) such as "Cytokine", "Growth factor" and "Secreted" in senescence-induced human pancreatic stellate cells (hPSCs) by the treatment with hydrogen peroxide.

Gene name
C-C motif chemokine ligand 19 (CCL19)
C-X-C motif chemokine ligand 1 (CXCL1)
C-X-C motif chemokine ligand 2 (CXCL2)
C-X-C motif chemokine ligand 3 (CXCL3)
TNF receptor superfamily member 10a (TNFRSF10A)
TNF receptor superfamily member 10c (TNFRSF10C)
TNF superfamily member 18 (TNFSF18)
interleukin 1 beta (IL1B)
interleukin 13 receptor subunit alpha 2 (IL13RA2)
interleukin 21 (IL21)
interleukin 24 (IL24)
interleukin 33 (IL33)
interleukin 6 receptor (IL6R)
platelet factor 4 variant 1 (PF4V1)
platelet factor 4 (PF4)
thymic stromal lymphopoietin (TSLP)
KIT ligand (KITLG)
Amphiregulin (AREG)
cellular communication network factor 3 (CCN3)
cerebral dopamine neurotrophic factor (CDNF)
growth differentiation factor 15 (GDF15)
insulin like growth factor 1 (IGF1)
interleukin 7 (IL7)
placental growth factor (PGF)
ADAM metallopeptidase with thrombospondin type 1 motif 5 (ADAMTS5)
FAM20A golgi associated secretory pathway pseudokinase (FAM20A)
Fc mu receptor (FCMR)
R-spondin 3 (RSPO3)
TIMP metallopeptidase inhibitor 4 (TIMP4)
adhesion G protein-coupled receptor G1 (ADGRG1)
aldo-keto reductase family 1 member B10 (AKR1B10)
amylase alpha 1B (AMY1B)
amylase alpha 1C (AMY1C)
carbonic anhydrase 11 (CA11)
carboxyl ester lipase (CEL)
carboxypeptidase Z (CPZ)
Cholecystokinin (CCK)
collagen type IV alpha 6 chain (COL4A6)
collagen type VI alpha 6 chain (COL6A6)
complement C1r subcomponent like (C1RL)
cystatin SA (CST2)
cystatin SN (CST1)
ectonucleotide pyrophosphatase/phosphodiesterase 2 (ENPP2)
fibronectin leucine rich transmembrane protein 2 (FLRT2)
folate receptor gamma (FOLR3)

laminin subunit beta 4 (LAMB4)
macrophage stimulating 1 like (pseudogene)(MST1L)
matrix metallopeptidase 1 (MMP1)
matrix metallopeptidase 12 (MMP12)
matrix metallopeptidase 3 (MMP3)
netrin 5 (NTN5)
ninjurin 1 (NINJ1)
papilin, proteoglycan like sulfated glycoprotein (PAPLN)
pappalysin 1 (PAPPA)
parathyroid hormone like hormone (PTHLH)
placenta enriched 1 (PLAC1)
Proenkephalin (PENK)
proline rich protein BstNI subfamily 2 (PRB2)
proline rich protein BstNI subfamily 3 (PRB3)
proline rich protein BstNI subfamily 4 (PRB4)
retinoic acid early transcript 1E (RAET1E)
secreted and transmembrane 1 (SECTM1)
secreted phosphoprotein 1 (SPP1)
synuclein alpha (SNCA)
tenascin N (TNN)
